# Two novel amino acid substitutions in highly conserved regions of prion protein (PrP) and a high frequency of a scrapie protective variant in native Ethiopian goats

**DOI:** 10.1186/s12917-019-1870-4

**Published:** 2019-05-03

**Authors:** Maria Vitale, Sergio Migliore, Berhanu Tilahun, Mukarim Abdurahaman, Marco Tolone, Ignazio Sammarco, Vincenzo Di Marco Lo Presti, Endrias Zewdu Gebremedhin

**Affiliations:** 1 Istituto Zooprofilattico Sperimentale of Sicily, Laboratory of Genetics of Microorganisms, Via Gino Marinuzzi 3, 90129 Palermo, Italy; 2 Department of Parasitology, Haramaya University, College of Veterinary Medicine, P.O. Box 138, Dire Dawa, Ethiopia; 3 Jimma University, School of Veterinary Medicine, P.O. Box 307, Jimma, Ethiopia; 4 Dipartimento Scienze Agrarie, Alimentari e Forestali, Università degli Studi di Palermo, Viale delle Scienze, 90128 Palermo, Italy; 5 Department of Veterinary Science, Ambo University, College of Agriculture and Veterinary Sciences, P.O. Box 19, Ambo, Ethiopia

**Keywords:** Ethiopian native goats; novel polymorphisms, *PRNP*, PrP, Scrapie

## Abstract

**Background:**

Polymorphisms of the prion protein gene may influence scrapie susceptibility in small ruminants through modified protein conformation. At least 47 amino acid substitutions and 19 silent polymorphisms have been described in goat *PRNP* reported from several countries. The objective of this study was to investigate *PRNP* polymorphisms of native Ethiopian goat breeds and compare the results with other goat breeds.

**Results:**

The analysis of the prion protein gene *PRNP* in 229 goats belonging to three of the main Ethiopian native goat breeds showed a remarkably high frequency (> 34.6%) of p.(Asn146Ser) in these breeds, a variant involved in scrapie resistance in Cyprus. In addition, two novel amino-acid substitutions p.(Gly127Ala) and p.(Thr193Ile), with frequencies ranging from 1.5 to 7.3% were detected. Both amino acids are well conserved in prion proteins (PrP) of most species and these changes have never been reported before in goats worldwide. Residue 127 is within the N–terminal domain of PrP and is probably involved in the recruitment of neural cell adhesion molecules (NCAM). Residue 193 is within the highly conserved string of 4 threonines that plays a role in determining the efficiency of prion protein conversion towards its pathological form.

**Conclusion:**

Two novel coding polymorphisms and a high frequency of a scrapie protective variant indicate a high level of genetic diversity in *PRNP* of Ethiopian goats. This finding increases the interest in exploring *PRNP* polymorphisms of native goat breeds in areas where cross breeding with foreign goats has rarely occurred.

**Electronic supplementary material:**

The online version of this article (10.1186/s12917-019-1870-4) contains supplementary material, which is available to authorized users.

## Background

Scrapie is a fatal, neurodegenerative disease that affects small ruminants world-wide. It belongs to the group of prion diseases or transmissible spongiform encephalopathies (TSEs), such as bovine spongiform encephalopathy (BSE) in cattle, chronic wasting disease (CWD) in cervids and Creutzfeldt–Jakob disease (CJD) in humans. Recently, a new prion disease, Camel Prion Disease (CPD) of unknown origin was detected in dromedary camels (*Camelus dromedarius*) in Algeria enlarging the spectrum of animal species susceptible to prion disease [1].

The conversion of normal prion protein (PrP) into pathogenic PrP conformers is central to prion diseases. Polymorphisms of the prion protein gene (*PRNP*) sequences may influence scrapie susceptibility in sheep and goats through modified protein conformation [2]. Although small changes in the amino acid sequence do not significantly alter the overall structure of the cellular PrP [3] they can severely alter prion transmissibility [4].

Goat *PRNP* displays at least 47 amino acid substitutions and 19 silent polymorphisms that were described in several countries [5]. The amino acid substitution p.(Gln222Lys) has been described as a protective allele in experimental and epidemiological studies in several European goat breeds [6–10]. An important factor in the genetic resistance to scrapie is related to the variants p.(Asn146Ser) or (Asn146Asp) which are described particularly on the island of Cyprus [11]. Recently, the impact of these variants on prion infectivity and disease outcome has been confirmed through experimental infections [12].

Ethiopia possesses about 29.1 million sheep and 29.3 million goats [13] raised in various agro-ecological zones. Goat production is economically and socially important and the native goats are widely distributed in all regions of the country in relation to environmental adaptation and survival in different areas. In lowland areas, goats are raised for milk and meat production, while in the highlands they are mainly kept for the meat. In addition, goats are also a source of non-food products such as skin for local leather industries [14]. Based on their phenotypic characteristics and genetic differences at the DNA level, four distinct families and 12 breeds of goats have been identified in Ethiopia [15, 16]. A family is a group of breeds that are genetically more related and physically more similar than breeds outside the group. Central Highland and Western Highland breeds of goat belong to the small East African family while the Long-eared Somali breed belongs to the Somali family [15, 16].

As is the case for most African countries, no surveillance for scrapie is performed and no history of TSE is available in Ethiopia. The objective of this study was to investigate *PRNP* polymorphisms of native Ethiopian goat breeds and to compare the results with available data reported in other goat breeds.

## Results

**Fig. 1:**
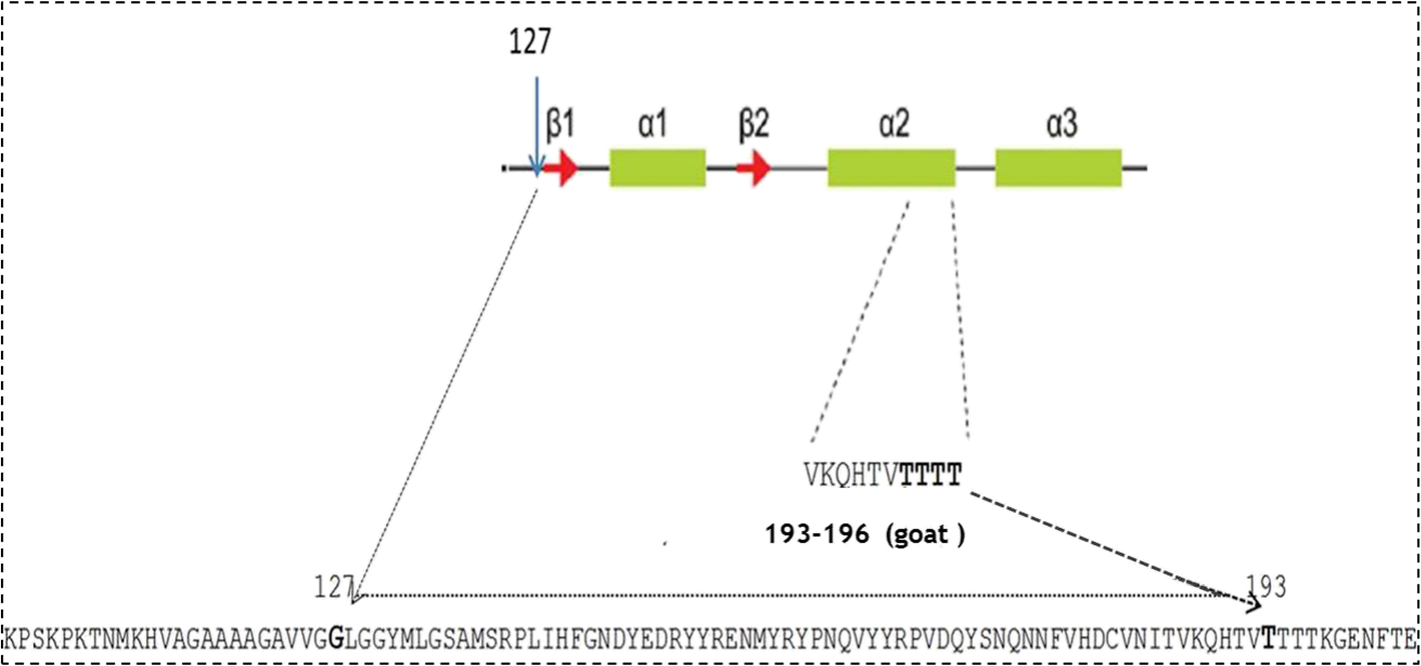
Schematic of PrP with the two novel PrP amino acid substitutions in bold. The green areas represent the 3 α-helixes; ß1 and ß2 (in red) are the ß-sheets of the PrP. The amino acid sequence of PrP corresponding to residues 104–206 are from the PrP of *Capra hircus* sequence (accession number P52113). The sequence is expressed with one letter code of each amino acid (see abbreviations list)

Two novel amino acid variations were detected in all Ethiopian goat breeds in two important and well-conserved regions of PrP (Fig. 1): p.(Gly127Ala) and p.(Thr193Ile). All detected genotypes are reported in Table 1. The allelic frequencies are reported in Table 2 and no statistically significant difference was found regarding their distribution between the analysed breeds (*P* > 0.05). The variant p.(Gly127Ala) showed a frequency of 4.1% in Western Highland goats and lower frequencies in Central Highland and Long-eared Somali goats respectively. The variant p.(Thr193Ile) showed higher frequencies in Western and Central Highland breeds and a lower frequency in the Long-eared Somali goat (Table 2). Additional four amino acid substitutions: p.(His143Arg), p.(Asn146Ser), p.(Arg154His), and p.(Ser240Pro) were detected. SNPs at codon 42 (ccg > cca) and codon 138 (agc > agt) resulted in silent protein changes p.(Pro42=) and p.(Ser138=). At codon 240 a higher frequency of proline compared to serine was observed in all Ethiopian breeds (Table 2) as in other goats worldwide [17]. The resistant allele p.(Asn146Ser), was observed in all breeds in both homozygous and heterozygous combinations. However p. (Ser146Ser) was found only in combination with p.(Pro240Pro) (Additional file 2). The other resistant variant p.(Asn146Asp) was not detected. The variant p.(Arg154His) was found in one Western Highland goat and four Long-eared Somali goats and in this latter breed, a single goat with p. (His143Arg) was also detected (Table 1).

**Table 1:** Frequency of all genotypes detected in Ethiopian goats

Genotypes	Western Highland (N) %	Central Highland (N) %	Long Eared Somali (N) %
p.(Gly127Gly)	(101) 91.82	(50) 96.15	(65) 97.01
p.(Gly127Ala)	(9) 8.18	(2) 3.85	(2) 2.99
p.(Ala127Ala)	–	–	–
p.(His143His)	(110) 100.00	(52) 100.00	(66) 98.51
p.(His143Arg)	–	–	(1) 1.49
p.(Arg143Arg)	–	–	–
p.(Asn146Asn)	(38) 34.55	(23) 44.23	(22) 32.84
p.(Asn146Ser)	(55) 50.00	(22) 42.31	(37) 55.22
p.(Ser146Ser)	(17) 15.45	(7) 13.46	(8) 11.94
p.(Arg154Arg)	(109) 99.09	(52) 100.00	(63) 94.03
p.(Arg154His)	(1) 0.91	–	(4) 5.97
p.(His154His)	–	–	–
p.(Thr193Thr)	(94) 85.46	(45) 86.54	(62) 92.54
p.(Thr193Ile)	(16) 14.54	(7) 13.46	(5) 7.46
p.(Ile193Ile)	–	–	–
p.(Ser240Ser)	(4) 3.64	(1) 1.92	(2) 2.99
p.(Ser240Pro)	(20) 18.18	(22) 42.31	(16) 23.88
p.(Pro240Pro)	(86) 78.18	(29) 55.77	(49) 73.13

**Table 2:** Allelic frequency calculated over the total genotypes of Ethiopian goats

						Western Highland (N)	Central Highland (N)	Long Eared Somali (N)	All (N)
p.(Gly127Gly)	p.(His143His)	p.(Asn146Asn)	p.(Arg154Arg)	p.(Thr193Thr)	p.(Ser240Ser)	0.104 (23)	0.135 (14)	0.104 (14)	0.111 (51)
p.(Gly127Ala)	–	–	–	–	p.(Ser240Pro)	0.041 (9)	0.019 (2)	0.015 (2)	0.028 (13)
–	p.(His143Arg)	–	–	–	p.(Ser240Pro)	0	0	0.007 (1)	0.002 (1)
–	–	p.(Asn146Ser)	–	–	p.(Pro240Pro)	0.405 (89)	0.346 (36)	0.396 (53)	0.389 (178)
–	–	–	p.(Arg154His)	–	p.(Ser240Pro)	0.005 (1)	0	0.030 (4)	0.011 (5)
–	–	–	–	p.(Thr193Ile)	p.(Pro240Pro)	0.073 (16)	0.067 (7)	0.037 (5)	0.061 (28)
–	–	–	–	–	p.(Pro240Pro)	0.372 (82)	0.433 (45)	0.410 (55)	0.397 (182)
						1.00 (220)	1.00 (104)	1.00 (134)	1.00 (458)

## Discussion

In this paper two novel coding polymorphisms and a relatively high frequency of p.(Asn146Ser) are reported for Ethiopian goat breeds. The novel variant p. (Gly127Ala) is located within the N terminal domain of PrP involved in the interaction with the neuronal cell adhesion molecule (NCAM) [18]. A variant with serine at the same position p.(Gly127Ser) was already described in Europe, America and Asia [5], and related to a delay in the clinical onset of the disease through epidemiological and experimental studies [19–21]. The other variant p.(Thr193Ile), is found within the highly conserved string of 4 threonines located at the C-terminus of the α2-helix of PrP conferring a high instability to the protein, with great propensity to form β-sheets [22]. The first threonine (T) of the string (Thr193 in goat), has been proposed to initiate the pathologic PrP conversion through an increased formation of fibrils [23]. A substitution in the same amino acid string with proline at codon 194 was already described in mixed Southern Italian goats [24] but its role in scrapie resistance or susceptibility remains unknown. A study on mouse PrP, reported a replacement with valine (V) at codon 189 (equivalent to codon 193 in goat) that prevents prion disease-related pathologic conversion by conferring more stability to the prion protein [25]. The two novel variants p.(Gly127Ala) and p.(Thr193Ile) detected in this study might have similar effects to the above-mentioned p.(Gly127Ser) in other goats and p.(Thr189Val) in mouse, since all are radical replacements. A high prevalence of proline at codon 240 was also detected as in other goat breeds worldwide [17] but amino acid variations at codon 240 have no impact on scrapie disease [26]. However the majority of variations in other codons were associated to p.(Ser240Pro) in this study (Additional file 2). The variant p.(Asn146Ser) was detected at a frequency of 40.5% in Western Highland goats, 34.6% in Central Highland goats and 39.6% in Long-eared Somali goats (Table 2). This scrapie protective variant has been described already in other native breeds in Tanzania at a frequency of 16.3% [27], and 28.1% in Halep goats in Turkey [28]. Frequencies similar to Ethiopian goats have been reported in imported Boer goats in Great Britain (24.5–35.5%) [29], in the USA (35.2%) [30], and in the Netherlands (31%) [31], suggesting that this resistance-associated polymorphism has been maintained in the Damascus-related breeds independently from the presence of scrapie [28]**.** However, serine at codon 146 is present at frequency of 16.5% in Damascus goat of Cyprus [32] but absent in Derivata di Siria, another Damascus related goat breed in Sicily, that showed conversely a 15% frequency of the resistant variant p.(Gln222Lys) [33]. Both islands are considered as endemic areas for scrapie, so it could be possible that different alleles in native breeds were positively selected in relation to the local circulating scrapie strains in specific geographic areas [34].

The presence of p.(Asn146Ser) in Halep (Damascus), Ethiopian and Tanzanian native goats suggests that the allele was naturally selected in the Middle East first, and spread through the central Sahara and Ethiopia later [35, 36] and subsequently into southern parts of the African continent through a different migration route compared to the main Mediterranean and North African routes. As in the Sicilian Damascus breed, p.(Asn146Ser) has not been detected so far in Algerian, Tunisian and Moroccan goats [37, 38]

The frequency of the protective variant p.(Asn146Ser) in three of the main Ethiopian goat breeds and the presence of two novel amino acid substitutions in highly conserved regions of prion protein (PrP), show a major diversity in *PRNP* of Ethiopian goats compared to other breeds. However, a low genetic variation among indigenous Ethiopian goats has been detected through genetic studies on microsatellites probably due to the ancient and widespread practice of nomadism [39, 40]. This traditional practice might also explain the presence of different goat families that overlap breeds and the similarity of *PRNP* substitution frequencies among the breeds in this study. In any case extensive cross breeding with goats coming from other countries has never been performed in Ethiopia, except for some pilot studies with Saanen goats to increase milk production [14], so these results reflect the original genetic background of Ethiopian goats.

## Conclusion

The two novel amino acid substitutions and the high frequency of the scrapie protective variant p.(Asn146Ser) might indicate a lower risk of scrapie in Ethiopian goats. It could be very important to perform *PRNP* characterization also in sheep populations of the same areas, especially because mixed holdings are very common. A surveillance plan for the disease in both goats and sheep populations would be also important to understand the true epidemiology of scrapie in the country. The occurrence of a new prion disease in dromedaries, CPD in Algeria, could be an impetus for a surveillance plan for prion diseases in Africa since dromedaries are often bred with sheep and goats simultaneously; sharing common pastures in many African countries such as in Ethiopia. A new prion disease in a farmed animal species requires a thorough risk assessment for implementing policies to control the disease in animals and minimize human exposure [1]. This study can fill gaps on the knowledge of *PRNP* polymorphisms of goats in economically marginal areas and increase the interest in exploring the genetic variation of the *PRNP* gene in native goat breeds and sheep as well, in countries where cross-breeding with imported animals has never been performed.

## Methods

### Goat breeds

Three of the main Ethiopian native goat breeds were analysed for the *PRNP* gene: Western and Central Highlands and Long-eared Somali. Somatic aspects of the different goats have been already described [15, 16, 41]. Professional veterinarians collected the blood samples aseptically from jugular veins using ethylene diamine tetraacetic acid (EDTA) coated tubes. A total of 229 goats, belonging to 80 herds were sampled. Central Highland breeds (*n* = 52) were sampled in the Seka, Tiroafata and Omonada districts of Jimma; Western Highland (*n* = 110) from the Ambo and Bako districts; and Long eared Somali *(n* = 67) from the Gursum and Jijiga districts of Eastern Hararghe. The samples consisted of 75% males and 25% females with no genetic relation.

### Genetic analysis

Polymerase chain reaction (PCR) amplification of the open reading frame of the caprine *PRNP* (NM_001314247.1 for the cDNA position and NP_001301176.1 for the protein variants) was performed and direct PCR fragment sequencing was carried out as previously described [42].

Raw data of Ethiopian goats genotyping are reported in Additional file 1

Amino acid variants are described following recommendation of Den Dunnen et al. [43]

Allelic frequencies were calculated using the Hardy-Weinberg equation. Statistical differences between the breeds were calculated with a chi-square test.

## Additional files

Additional file 1: Prion protein gene polymorphism in native Ethiopian goats. Excel file that include all information related to each single goat in the three Ethiopian regions. For each goat, information regarding sex, age in months and the related prion polymorphisms are reported. (XLS 60 kb)

Additional file 2: Table of the genotypes combinations of Prion protein gene in native Ethiopian goats.. (PDF 423 kb)

## Abbreviations

BSE: Bovine spongiform encephalopathy
CWD: Chronic wasting disease
*PRNP*: Prion protein gene
PrP: Prion protein
TSE: Transmissible spongiform encephalopathy

## Amino acid code

Ala (A): Alanine
Arg (R): Arginine
Asn (N): Asparagine
Asp (D): Aspartic acid
Gln (Q): Glutamine
Gly (G): Glycine
His (H): Histidine
Ile (I): Isoleucine
Lys (K): Lysine
Pro (P): Proline
Ser (S): Serine
Thr (T): Threonine
Val (V): Valine
