# Supplementary material for: Two novel amino acid substitutions in highly conserved regions of prion protein (PrP) and a high frequency of a scrapie protective variant in native Ethiopian goats
Source: BMC Vet Res. 2019 May 3;15:128. doi: 10.1186/s12917-019-1870-4 (PMC6500044; doi:10.1186/s12917-019-1870-4)
Supplement: Supplementary file 2 — Table of the genotypes combinations of Prion protein gene in native Ethiopian goats.. (PDF 423 kb) [file 12917_2019_1870_MOESM2_ESM.pdf]

Table 3:Genotype combinations

127 G/A	143 H/R	146 N/S	154 R/H	193 T/I	240 S/P	Western Highland % (N)	Central Highland % (N)	Long Eared Somali % (N)	All % (N)
GG	HH	NN	RR	TT	SS	3.6 (4)	1.9 (1)	3.0 (2)	**3.06 (7)**
-	-	-	-	-	PP	12.8 (14)	17.3 (9)	14.9 (10)	**14.41 (33)**
-	-	-	-	-	SP	9.1 (10)	15.3 (8)	6.0 (4)	**9.61 (22)**
GA	-	NS	-	-	PP	3.6 (4)	1.9 (1)	3.0 (2)	**3.06 (7)**
GA	-	-	-	-	PP	1.8 (2)	1.9 (1)	0	**1.31 (3)**
GA	-	-	-	-	SP	0.9 (1)	0	0	**0.44 (1)**
GA	-	-	-	TI	PP	1.8 (2)	0	0	**0.87 (2)**
-	HR	-	-	-	SP	0	0	1.5 (1)	**0.44 (1)**
-	-	NS	-	-	PP	29.1 (32)	13.5 (7)	35.8 (24)	**27.51 (63)**
-	-	NS	-	-	SP	8.2 (9)	21.2 (11)	11.9 (8)	**12.23 (28)**
-	-	NS	RH	-	SP	0	0	1.5 (1)	**0.44 (1)**
-	-	NS	-	TI	PP	9.1 (10)	5.8 (3)	3.0 (2)	**6.55 (15)**
-	-	SS	-	-	PP	15.5 (17)	13.5 (7)	11.9 (8)	**13.97 (32)**
-	-	-	RH	-	PP	0.9 (1)	0	1.5 (1)	**0.87 (2)**
-	-	-	RH	-	SP	0	0	1.5 (1)	**0.44 (1)**
-	-	-	RH	TI	SP	0	0	1.5 (1)	**0.44 (1)**
-	-	-	-	TI	PP	3.6 (4)	1.9 (1)	3.0 (2)	**3.06 (7)**
-	-	-	-	TI	SP	0	5.8 (3)	0	**1.31 (3)**
						**100 (110)**	**100 (52)**	**100 (67)**	**100 (229)**
